# Supplementary material for: The genome sequence of kidney vetch, Anthyllis vulneraria L. (Fabales: Fabaceae)
Source: Wellcome Open Res. 2026 Sep 17;11:687. [Version 1] doi: 10.12688/wellcomeopenres.27830.1 (PMC13650319; doi:10.12688/wellcomeopenres.27830.1)
Supplement: Peer review history

# Reviewer report: Reviewer response for version 1

**DOI:** 10.21956/wellcomeopenres.30625.r172344

**Published:** 2026-10-16

**Authors:** Martin Laforest

**Affiliations:** 1 Research and Development Centre, Agriculture and Agri-Food Canada, Saint-Jean-sur-Richelieu, Quebec, Canada

Drs Ruhsam, Hollingsworth and several teams from Wellcome Sanger Institute Tree of Life present the genome sequence of kidney vetch (Anthyllis vulneraria L.). This research article is well written, clear and provides a detailed presentation of the experimental and analytic procedures to determine the genome sequence of this legume.

 There are three very minor points I would like to report. First, I think it would be of interest to provide the meaning of the word “Wundklee” [ *Wunde* (wound) and *Klee* (clover)]. Second, the acronym WSI appears before (bottom of page 4) it is presented in full (top of page 5). Finally, I could not find clearly where the threshold used for the GQN analysis was mentioned. For consistency, it would be nice to have this value for the 3 libraries.

Are the datasets clearly presented in a usable and accessible format, and the assembly and annotation available in an appropriate subject-specific repository?

Yes

Are sufficient details of the sequencing and extraction, software used, and materials provided to allow replication by others?

Yes

Are the rationale for sequencing the genome and the species significance clearly described?

Yes

Are the protocols appropriate and is the work technically sound?

Yes

Reviewer Expertise:

Weed Science, molecular mechanisms of herbicide resistance

I confirm that I have read this submission and believe that I have an appropriate level of expertise to confirm that it is of an acceptable scientific standard.

# Reviewer report: Reviewer response for version 1

**DOI:** 10.21956/wellcomeopenres.30625.r169683

**Published:** 2026-10-13

**Authors:** Marco Todesco

**Affiliations:** 1 University of British Columbia, Vancouver, Canada

This genome note presents a genome assembly for kidney vetch ( *Anthyllis vulneraria*) as part of the Darwin Tree of Life (DToL) project. The authors provide a comprehensive introduction to the species, which makes a good case for the value of this assembly in resolving taxonomic relationships within the species complex.

 The assembly is, as expected for DToL, of very high quality. I would have liked some more information on why it was necessary to produce three separate HiFi libraries for this species, given its small genome.

 The Hi-C heatmap appears to show relatively few long-range information supporting chromosome scaffolding (compared with the background of inter-chromosomal interactions) - I am wondering if that is due to difficulties constructing Hi-C libraries (it looks like the vast majority of interactions are very local).

Are the datasets clearly presented in a usable and accessible format, and the assembly and annotation available in an appropriate subject-specific repository?

Yes

Are sufficient details of the sequencing and extraction, software used, and materials provided to allow replication by others?

Yes

Are the rationale for sequencing the genome and the species significance clearly described?

Yes

Are the protocols appropriate and is the work technically sound?

Yes

Reviewer Expertise:

plant genetics, adaptation, genomics

I confirm that I have read this submission and believe that I have an appropriate level of expertise to confirm that it is of an acceptable scientific standard.

# Reviewer report: Reviewer response for version 1

**DOI:** 10.21956/wellcomeopenres.30625.r172337

**Published:** 2026-10-08

**Authors:** Ruslan Kalendar

**Affiliations:** 1 University of Helsinki, Helsinki, Finland

The authors present a phased assembly of the diploid (2n = 12) genome of *Anthyllis vulneraria* generated within the Darwin Tree of Life project, using PacBio HiFi (42.33 Gb; Sequel IIe and Revio), Hi-C (131.14 Gb) and RNA-seq data. Haplotype 1 (334.47 Mb) has been curated to chromosome level: six pseudomolecules contain 92.49% of the sequence, with contig N50 2.73 Mb, scaffold N50 50.15 Mb, QV 59.9 and BUSCO completeness of 98.0%. Haplotype 2 (342.59 Mb) remains at scaffold level. The plastome (151.91 kb) and three mitochondrial molecules were also assembled.

 The assembly is of high quality and will be a useful resource.  

 Q1. Flow cytometry gave 1C = 0.43 pg (420 Mb), GenomeScope 350.97 Mb, haplotype 1 334.47 Mb (80% of the FCM value), and haplotype 2 342.59 Mb. The sentence “These estimates guided expectations for the assembly” does not explain a shortfall of 75-85 Mb. With an estimated repeat content of 49.8%, collapsed tandem repeats (satellites, centromeric arrays, 45S/5S rDNA) are the likely explanation.

 Q2. The text reports “approximately 112× coverage of the haplotype 1 assembly,” but 42.33 Gb / 334.47 Mb = 126.6×. In the blob plot (Figure 6), the chromosomal scaffolds sit at approximately 60×. Presumably, 112× corresponds to the homozygous k-mer peak in GenomeScope (2 × kcov 56.1), while the blob plot may be based on a subset of reads.

 Q3. Table 5 gives the BUSCO benchmark as “S > 90%; D < 5%”; however, haplotype 1 has D = 5.6% and haplotype 2 D = 5.0% (not below 5%). The k-mer completeness of haplotype 1 (89.20%) is also below the 90% threshold; this is expected for a single haplotype at 0.58% heterozygosity, but it should be noted. The duplicated BUSCOs deserve a comment: residual haplotypic duplication versus genuine paralogues (e.g., from the ancient papilionoid whole-genome duplication).

 Q4. According to Figure 6, 487 scaffolds totalling 25.4 Mb were classified as “no-hit”. They form distinct clusters: GC 52-54% at 50-500× coverage (probably rDNA or satellite repeats), and GC 56-58% at low coverage of 1-10× (residual contamination or ULI-library artifacts cannot be excluded).

 Q5. The Methods section states that both haplotypes were combined for curation; however, haplotype 2 remains at the scaffold level even though it is longer (342.59 Mb) and its scaffold N50 (48.72 Mb) and longest scaffold (88.86 Mb) are essentially chromosome-scale. Please explain why haplotype 2 was not curated to chromosome level and on what basis haplotype 1 was chosen as the reference.

Are the datasets clearly presented in a usable and accessible format, and the assembly and annotation available in an appropriate subject-specific repository?

Yes

Are sufficient details of the sequencing and extraction, software used, and materials provided to allow replication by others?

Yes

Are the rationale for sequencing the genome and the species significance clearly described?

Yes

Are the protocols appropriate and is the work technically sound?

Yes

Reviewer Expertise:

plant genome bioinformatics

I confirm that I have read this submission and believe that I have an appropriate level of expertise to confirm that it is of an acceptable scientific standard.

# Reviewer report: Reviewer response for version 1

**Published:** 2026-09-17

**Authors:** Automated Benchmarking

**Affiliations:** 1 Wellcome Sanger Institute, Hinxton, UK

As this article is a Genome Note, Automated Benchmarking has been used to determine whether the reported assembly passes four defined checks established by the Earth BioGenome Project: the raw and assembled data are publicly available, the Earth BioGenome Project three-part metric is met, the k-mer completeness exceeds 90%, and the BUSCO score passes the stated threshold. For Anthyllis vulneraria (drAntVuln1), all four checks passed. Data available: pass. ENA study PRJEB76526, raw-read accessions ERR13265040, ERR13265038, ERR13265039, ERR13301070, and ERR13962517, and assembly accessions GCA_965615755.1 and GCA_965615695.1 are public. Earth BioGenome Project three-part metric: pass. Haplotype 1 is reported as 6.C.Q59, meeting the 6.C.Q40 reference standard. k-mer completeness: pass. The combined haplotype assemblies have 98.59% k-mer completeness, exceeding the Earth BioGenome Project target of 90%. BUSCO: pass. Haplotype 1, which is proposed as the reference assembly for this species, has 98.0% complete BUSCOs (92.4% single-copy and 5.6% duplicated). BUSCO software version: 6.0.0; lineage dataset: fabales_odb10. It meets the plant-specific target of greater than 90% complete BUSCOs. This decision uses values reported in the Genome Note and public accession records.

Reviewer Expertise:

NA

I confirm that I have read this submission and believe that I have an appropriate level of expertise to confirm that it is of an acceptable scientific standard.
